# Nitric oxide synthases in infants and children with pulmonary hypertension and congenital heart disease

**DOI:** 10.1186/1465-9921-10-110

**Published:** 2009-11-13

**Authors:** Thomas Hoehn, Brigitte Stiller, Allan R McPhaden, Roger M Wadsworth

**Affiliations:** 1 Neonatology and Pediatric Intensive Care Medicine, Department of General Pediatrics, Heinrich-Heine-University, Duesseldorf, Germany; 2 Department of Congenital Heart Disease, University Hospital, Freiburg and Department of Pediatric Cardiology, Deutsches Herzzentrum, Berlin, Germany; 3 Department of Pathology, Glasgow Royal Infirmary, Glasgow, UK; 4 Department of Physiology and Pharmacology, University of Strathclyde, Glasgow, UK

## Abstract

**Rationale:**

Nitric oxide is an important regulator of vascular tone in the pulmonary circulation. Surgical correction of congenital heart disease limits pulmonary hypertension to a brief period.

**Objectives:**

The study has measured expression of endothelial (eNOS), inducible (iNOS), and neuronal nitric oxide synthase (nNOS) in the lungs from biopsies of infants with pulmonary hypertension secondary to cardiac abnormalities (n = 26), compared to a control group who did not have pulmonary or cardiac disease (n = 8).

**Methods:**

eNOS, iNOS and nNOS were identified by immunohistochemistry and quantified in specific cell types.

**Measurements and main results:**

Significant increases of eNOS and iNOS staining were found in pulmonary vascular endothelial cells of patients with congenital heart disease compared to control infants. These changes were confined to endothelial cells and not present in other cell types. Patients who strongly expressed eNOS also had strong expression of iNOS.

**Conclusion:**

Upregulation of eNOS and iNOS occurs at an early stage of pulmonary hypertension, and may be a compensatory mechanism limiting the rise in pulmonary artery pressure.

## Introduction

Nitric oxide (NO) plays a central role in the maintenance of normal pulmonary vascular tone and healthy lung function [1]. All 3 isoforms of nitric oxide synthase (NOS) are present in the lungs and contribute to NO production in specific cell types [2]. Pediatric pulmonary disease is associated with endothelial dysfunction and consequently reduced NO delivery from the pulmonary vascular endothelium [3]. Moreover there is evidence from experimental models of neonatal pulmonary hypertension that impairment of NOS can generate reactive oxygen species, leading to a further cycle of deterioration of the vascular endothelium [4]. In adults with pulmonary arterial hypertension it has been demonstrated that output of NO is diminished [5], and that those patients who responded well to therapy had corresponding improvement in exhaled NO [6]. NO status can be improved by administration of inhaled NO which is valuable in the management of infants with pulmonary hypertension [7-10].

We chose to immunohistochemically investigate changes in NOS expression during the early course of pulmonary hypertension. Studies with experimental models of pulmonary hypertension have shown upregulation of endothelial NOS (eNOS) in the endothelial layer of both large and small pulmonary arteries [11]. Increased expression of eNOS was due to the initiating stimulus (hypoxia) and was not secondary to hyperperfusion [12]. The upregulation of eNOS correlated in time with the development of pulmonary hypertension [13]. In cultured pulmonary endothelial cells, acute exposure to hypoxia also upregulated eNOS [14]. There are several molecular mechanisms through which hypoxia can stimulate eNOS accumulation in endothelial cells, including hypoxia inducible factor [15] and phosphorylated cyclic-AMP response element binding protein (pCREB) [16]. Others have shown decreased expression of eNOS during chronic hypoxia in rats [17] and in human endothelial cells [18]. However in patients with pulmonary hypertension, it is less clear what changes in NOS isoform levels occur. In infants with congenital diaphragmatic hernia, it has been reported that pulmonary endothelium levels of iNOS were decreased [19] or unchanged [20], and similarly that pulmonary vascular endothelium levels of eNOS were decreased [21] or unaltered [19,20]. In adults with primary or secondary pulmonary hypertension, eNOS was reduced in the endothelial layer of small pulmonary arteries [22,23] but increased in plexiform lesions [22]. Given that the clinical studies have used patients with advanced disease whereas the experimental animal studies looked at an early stage of relatively mild pulmonary hypertension, we hypothesised that eNOS is raised initially when pulmonary hypertension is developing but falls at a late stage when endothelium dysfunction becomes severe. The aims of the present study were therefore to immunohistochemically determine the expression of the three isoforms of NOS in the lungs of infants with secondary pulmonary hypertension since they will have been exposed to elevated pulmonary pressure for a relatively short time and may therefore reveal what happens during the development of pulmonary hypertension.

## Methods

### Patients

Patients (n = 26) had a mean age of 16.9 months (± SEM = 4.02, median = 11 months, range: 2 months to 7 years) and had cardiac surgery performed between December 1985 and October 1991 at the German Heart Institute, Berlin, Germany. All patients had congenital cardiac defects typically associated with pulmonary hypertension and had a lung biopsy taken during corrective cardiac surgery. Surgery markedly reduced systolic pulmonary artery pressure with further reduction at follow up in patients, from whom data were available (for patient details see Table 1). Informed consent was obtained from the infants' parents, and the study protocol had previously been approved by the local institutional ethics committee.

**Table 1:** Patient details

dob	sex	Systolic PA-pressure pre-surgery	PVR dyn	Systolic PA-pressure post-surgery	Systolic PA-pressure after 6-36 months	Qp:Qs	Rp:Rs	Diagnosis	Age at surgery (months)	Heath + Edwards	Rabinovich
26.12.1990	f	75	1178	24	17	7,2	0,01	complete atrio-ventricular septal defect ventricular septal defect, atrial septal	5	2	a
28.03.1991	f		424			5	0,08	defect	6	2	b
20.01.1985	m		388			3	0,10	ventricular septal defect ventricular septal defect, atrial septal	11	2	b
20.03.1984	m		160			1,6	0,11	defect	60	1	a
20.10.1987	f		968			3,9	0,15	ventricular septal defect	12	2	c
03.12.1982	m	83	294	60	11	2,9	0,24	complete atrio-ventricular septal defect	84	1	c
12.08.1988	f	100	2400	30	22	2,6	0,27	complete atrio-ventricular septal defect ventricular septal defect, patent ductus	7	2	c
27.02.1991	f		1425			3,4	0,29	arteriosus, coarctation ventricular septal defect, atrial septal	6	1	a
25.11.1988	f		1855			2,8	0,30	defect	5	1	b
17.01.1985	f	80	2059	25	30	1,5	0,32	complete atrio-ventricular septal defect	14	0	0
14.06.1988	f	75	2222	25	14	2,1	0,32	complete atrio-ventricular septal defect single vessel disease, partial anomalous	5	1	b
09.03.1984	f		1285			1,9	0,33	pulmonary venous drainage	48	2	c
25.05.1990	m		2536			0,71	0,40	thoracic aortic constriction double-outlet right ventricle, ventricular	2	1	b
17.10.1980	m		717			1,9	0,40	septal defect, coarctation	11	3	c
06.04.1987	m		982			2,1	0,40	complete atrio-ventricular septal defect	19	1	c
15.05.1990	m		1883	35		2,3	0,41	ventricular septal defect	7	2	b
13.05.1988	f		1509			1,8	0,43	ventricular septal defect	11	2	0
10.02.1988	f	90	2061	38	18	1,8	0,45	complete atrio-ventricular septal defect	12	2	0
18.05.1990	f		3593			0,83	0,47	complete atrio-ventricular septal defect	4	1	a
22.09.1988	m		3537			1,2	0,50	atrial septal defect, patent ductus arteriosus	2	1	b
31.10.1989	m		2166			1,5	0,52	ventricular septal defect	11	1	b
03.11.1989	m		2617			1,4	0,71	mitral incompetence	11	2	a
05.04.1987	m	100	2135	25	35	1,6	0,71	ventricular septal defect	30	0	?
06.10.1984	f	93	983	75	34	1	0,83	ventricular septal defect	48	4	c
24.10.1988	f	83	2143	35	14	1,5	0,83	complete atrio-ventricular septal defect	6	2	b
25.05.1988	f	110	1888	40		1,3	0,90	ventricular septal defect	3	4	?

(CAVSD: complete atrio-ventricular septal defect; ASD: atrial septal defect; VSD: ventricular septal defect; MI: mitral incompetence); n = 26

### Control subjects

Control infants (n = 8) were chosen from infants and children having died from various non-pulmonary causes, who had an autopsy performed at the Department of Paidopathology, Humboldt University Berlin, Germany. None of these patients had clinical or echocardiographic evidence of pulmonary hypertension nor was there any clinical or radiologic evidence of pulmonary infection. Controls had a mean age of 7.1 months (± SEM = 1.75, median: 6 months, range: 2 to 17 months). For control details see Table 2.

**Table 2:** Controls

dob	sex	Age at death (months)	Diagnosis	PH
21.10.1992	f	5	Pulmonary stenosis	no

21.12.1991	m	17	D-transposition of the great arteries	no

04.08.1993	m	2	Hypoplastic left heart syndrome	no

06.04.1993	f	9	Mitochondriopathy	no

28.06.1993	m	10	Sudden infant death syndrome	no

20.07.1995	m	7	Carnitine-Palmitoyl-Transferase-Defect Type I	no

07.04.1996	f	2	Sudden infant death syndrome	no

22.04.1996	f	5	Omenn syndrome	no

(d-TGA: d-transposition of the great arteries; HLHS: hypoplastic left heart syndrome; SIDS: sudden infant death syndrome; CPT-defect: Carnitine-Palmitoyl-Transferase-Defect); n = 8

### Methodology for immunohistochemistry

Lung tissue was supplied as paraffin-embeded tissue blocks. Sections (4 μm) were cut from the blocks, rehydrated and then treated for antigen retrieval by microwave pressure cooking or trypsin incubation. The sections were then treated to block non-specific binding of primary and secondary antibodies and non-specific reaction with chromogens as described previously [11]. Sections were then incubated with the specific antibody for 60 minutes at room temperature (eNOS: catalogue reference 610296, BD Biosciences, UK, used at 1:1000 dilution along with pressure cooking antigen retrieval; iNOS: catalogue reference 610328, BD Biosciences, UK, used at 1:500 dilution along with pressure cooking antigen retrieval; nNOS: catalogue reference 610308, BD Biosciences, UK, used at 1:400 dilution along with trypsin antigen retrieval). Bound antibody was detected using goat anti-mouse IgG conjugated with horseradish peroxidase using a streptavidin-biotin link, and visualized with diaminobenzidine. In negative controls the primary antibody was replaced with pre-immune serum. Sections were counterstained using hematoxylin and viewed by light microscopy.

Staining intensity was quantified as follows: 0 = negative; 0.5 = faint/blush; 1 = mild; 2 = moderate. Separate quantification was performed for eNOS in small artery endothelium, small artery media, respiratory epithelium, alveolar macrophages. Antibody dilutions were chosen in order to differentiate between groups i.e. although there is usually baseline expression of eNOS in controls; dilutions were titrated until there was no eNOS expression visible in controls. For iNOS and nNOS, quantification was carried out in the same cell types except that alveolar macrophages and alveolar lining cells were combined. Vessels of an internal diameter of less than 250 μm were regarded as small pulmonary arteries.

### Statistics

For each antibody and cell type, the staining intensity of the cardiac patients was compared to the staining intensity of the normotensive patients using the Mann-Whitney-U test. Spearman's correlation coefficient has been calculated to describe the correlation between eNOS and iNOS expression. Statistical significance was assumed at p < 0.05.

## Results

**Figure 1:**
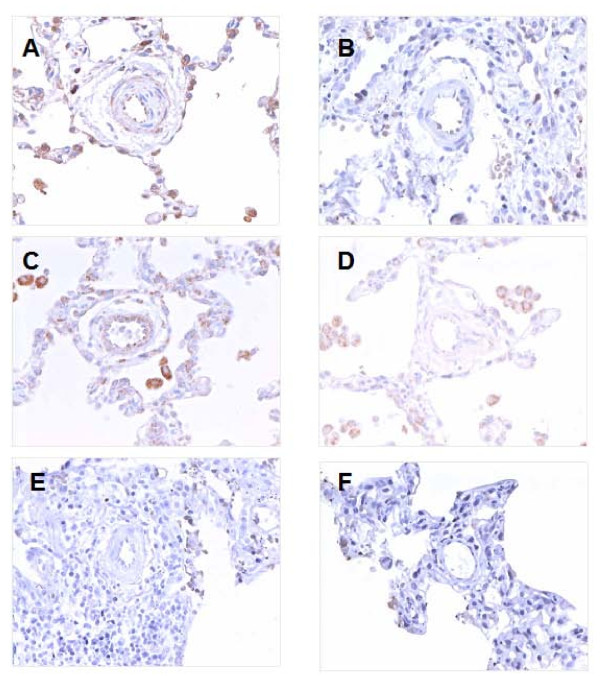
**Lungs from infants with pulmonary hypertension (A, C, E) and from control patients of similar age (B, D, F) stained for (A and B) eNOS, (C and D) iNOS, (E and F) nNOS**. (A) Cardiac patient small pulmonary artery showing mild endothelial positivity for eNOS. Intra-alveolar macrophages and alveolar lining cells also positive with very mild positivity also noted in media. (B) Small pulmonary artery from control patient showing very mild endothelial positivity for eNOS. (C) Cardiac patient small pulmonary artery showing iNOS positivity in endothelium and media. Intra-alveolar macrophages stained also strongly positive. (D) Small pulmonary artery of control patient showing no significant iNOS positivity. Intra-alveolar macrophages were positive. (E) Cardiac patient small pulmonary artery showing no immunocytochemical positivity for nNOS. (F) Control patient small pulmonary artery showing no positivity by immunocytochemistry for nNOS. × 400.

**Figure 2:**
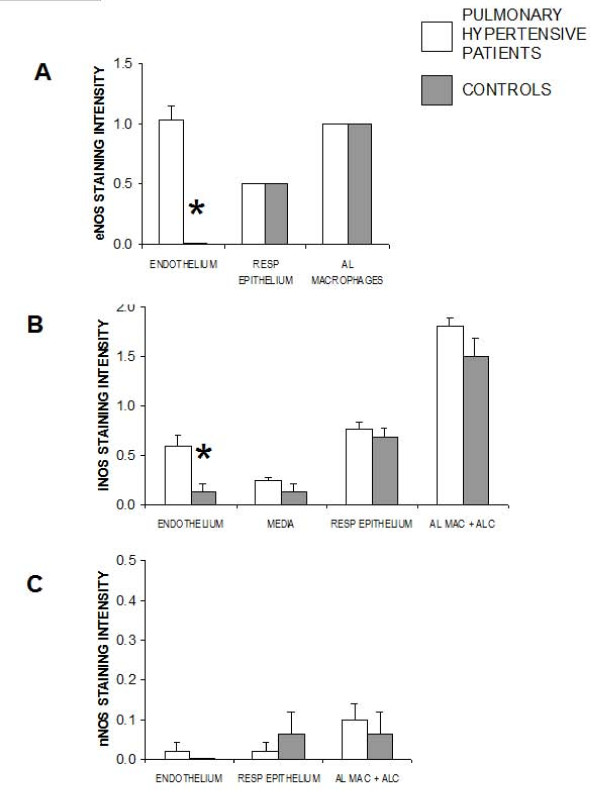
**Intensity of staining for (A) eNOS, (B) iNOS and (C) nNOS in lungs from infants with pulmonary hypertension and from control patients of similar age**. (A) Staining for eNOS was quantified separately in pulmonary vascular endothelium, respiratory endothelium, and alveolar macrophages of controls and cardiac patients (*p = 0.0001 comparing cases to controls). (B) Staining for iNOS was quantified in pulmonary vascular endothelium, pulmonary vascular media, respiratory endothelium, and alveolar macrophages/alveolar lining cells of controls and cardiac patients (* p = 0.008 comparing cases to controls). (C) Staining intensity for iNOS was quantified in pulmonary vascular endothelium, respiratory endothelium, and alveolar macrophages/alveolar lining cells of controls and cardiac patients. n = 23 cases, n = 8 controls.

In all of the lung sections from infants with pulmonary hypertension, thickening of the small pulmonary arteries was evident. In contrast there were no abnormalities of the pulmonary arteries in any normotensive control patients. There was expression of eNOS in the endothelial layer of small pulmonary arteries, the respiratory epithelium, and alveolar macrophages. Expression of eNOS was greatly increased in pulmonary hypertensive lungs compared to control lungs in the pulmonary artery endothelium (Figure 1, Figure 2). However there were no significant differences between controls and patient groups in staining for eNOS in alveolar macrophages and in the respiratory epithelium. Expression of iNOS was found in the small pulmonary arteries, both media and endothelium, the respiratory epithelium, and in alveolar macrophages/alveolar lining cells. There was significant upregulation of iNOS in endothelial cells of pulmonary hypertensive patients compared to control patients, but there were no differences between the cases and controls at any of the other cell types where iNOS was found (Figure 1, Figure 2). Expression of nNOS was very light in all cell types in the lung and was not different between cases and controls (Figure 1, Figure 2).

There was a significant correlation of eNOS and iNOS staining intensity in the pulmonary artery endothelium, such that patients having stronger staining in eNOS also had higher levels of iNOS (Spearman's correlation coefficient 0.72, p = 0.0004).

## Discussion

Here we report the consistent finding of an increase in eNOS expression during conditions of increased pulmonary vascular resistance secondary to congenital heart disease in infants and children. This upregulation appears to be linked to pulmonary hypertension in that it occurs in the pulmonary artery endothelium, but not in other sites where eNOS is present and nor is there any change in nNOS. We have previously shown increased expression of eNOS in pulmonary endothelial cells in infants with persistent pulmonary hypertension of the newborn (PPHN) [24] and in congenital pulmonary lymphangiectasis [25].

Previous studies of NOS enzyme expression in patients with pulmonary hypertension have examined either adults with severe pulmonary hypertension of many years' duration, or infants with congenital diaphragmatic hernia who have very severe hypertension. Patients with pulmonary hypertension classified as irreversible have been shown to have higher levels of eNOS expression, particularly in areas of severe vascular lesions [26]. Others found isolated increases in iNOS immunoreactivity but no changes in eNOS immunoreactivity in patients with congenital heart disease and flow-associated pulmonary hypertension [27]. The present study shows upregulation of eNOS and iNOS at an early stage of pulmonary hypertension, in agreement with the rat hypoxic model [11] and in contrast to published studies of end stage disease in pulmonary hypertensive patients [21-23]. This finding is consistent with the hypothesis that increased eNOS is associated with the initiation of pulmonary hypertension (chronic hypoxic model in rats and infants with pulmonary hypertension secondary to cardiac abnormalities) whereas at a late stage there is severe damage to the endothelium resulting in loss of eNOS. The decrease of eNOS expression with longstanding disease in adulthood [23] can be interpreted as the result of secondary damage to the pulmonary vasculature caused by a prolonged period of pulmonary hypertension, resulting in a failing endothelium with reduced production of NO. Additionally there may be other differences between infants and adult patients other than the duration of pulmonary hypertension which may have subtle effects on NOS expression.

The importance of NOS is demonstrated by the finding that mice with eNOS deletion have pulmonary hypertension [28]. However studies of animals that have either deletion or over-expression of eNOS and iNOS reveal that the physiological consequences of alterations in NOS abundance are complex. As expected, agonist contractions and HPV were both inhibited by gene delivery of either iNOS or of eNOS [29,30], however surprisingly there was no improvement in endothelium-dependent pulmonary relaxation [29]. Deletion of eNOS gene was associated with increased pulmonary artery muscularity, right ventricular hypertrophy and right ventricular pressure, but only in male and not in female mice [31]. Deletion of iNOS was not associated with evidence of pulmonary hypertension [31], however iNOS transfected mice had increased expression lasting only 7 days [30] making these experiments hard to interpret. Since eNOS deletion mice had upregulation of iNOS [28] it is clear that expression patterns of NOS isoforms are coupled. Thus the over-expression of eNOS and iNOS that we found in infants with pulmonary hypertension suggests but does not prove that this is a compensatory mechanism limiting the rise in pulmonary artery pressure. It is of interest that in our study patients with the more extreme upregulation of eNOS also had greater upregulation of iNOS, suggesting that changes in both isoforms are linked in the process of adaptation to pulmonary hypertension.

Our present data indicate that upregulation of eNOS is not a short term effect as might be anticipated in cases of PPHN. Rather can this increased expression of eNOS persist over months and years as shown in our oldest patients at the age of 5 and 7 years, respectively (Table 1).

Limitations of this study include the lack of enzyme activity data and the subjectivity of the immunohistochemical findings. We have consequently minimized the effect of confounding factors on the immunohistochemical data by applying strict protocols of quantification of staining intensity. The advantage of immunohistochemical studies is the microtopographic localization of the protein under investigation, which we regard as very important for the specific question of our study. Although protein activity studies would further strengthen the results of our investigation, unfortunately we had only paraffin blocks of lung tissue available thus preventing further protein activity studies.

In summary, we have shown upregulation of eNOS and iNOS in pulmonary endothelial cells at an early stage of pulmonary hypertension in infants with congenital heart disease. Additionally there is co-expression of these two enzymes in pulmonary endothelial cells of these infants. These findings support the hypothesis that infant pulmonary hypertension is different from adult disease and potentially more amenable to the therapeutic effect of anti-proliferative medication and thus prevention of early endstage pulmonary vascular disease.

## Competing interests

The authors declare that they have no competing interests.

## Authors' contributions

BS gathered the clinical data of the patients. ARM quantified the immunohistochemical staining. RMW and TH conceived the study, performed the statistical analysis and wrote the manuscript. All authors read and approved the final manuscript.
